# Measuring capital in active addiction and recovery: the development of the strengths and barriers recovery scale (SABRS)

**DOI:** 10.1186/s13011-020-00281-7

**Published:** 2020-06-16

**Authors:** David Best, Wouter Vanderplasschen, Mulka Nisic

**Affiliations:** 1 Business, Law and Social Sciences, University of Derby, One Friar Gate Square, Derby, Derbyshire DE1 1DF UK; 2 Department of Special Needs Education, University of Ghent, Henri Dunantlaan 2, B-9000 Ghent, Belgium; 3 Recovered Users Network, Rue Archimede 17, 1000 Brussels, Belgium

**Keywords:** Recovery, Recovery capital, Life in recovery, Assessment, Reintegration

## Abstract

**Background:**

The international Life In Recovery (LiR) surveys have provided an important message to the public and policy makers about the reality of change from addiction to recovery, consistently demonstrating both that there are marked gains across a range of life domains and that the longer the person is in recovery the better their recovery strengths and achievements. However, to date, no attempt has been made to quantify the Life In Recovery scales and to assess what levels of change in removing barriers and building strengths is achieved at which point in the recovery journey.

**Methods:**

The current study undertakes a preliminary analysis of strengths and barriers from the Life in Recovery measure, using data from a European survey on drug users in recovery (*n* = 480), and suggests that the instrument can be edited into a Strengths And Barriers Recovery Scale (SABRS). The new scale provides a single score for both current recovery strengths and barriers to recovery.

**Results:**

The resulting data analysis shows that there are stepwise incremental changes in recovery strengths at different recovery stages, but these occur with only very limited reductions in barriers to recovery, with even those in stable recovery typically having at least two barriers to their quality of life and wellbeing. Greater strengths in active addiction are associated with greater strengths and resources in recovery.

**Conclusion:**

As well as demonstrating population changes in each of the domains assessed, the current study has shown the potential of the Life In Recovery Scale as a measure of recovery capital that can be used to support recovery interventions and pathways.

## Introduction

Although recovery remains a contested term, there is a general agreement that this is a complex phenomenon that is individual and that occurs over time (Betty Ford Consensus Group [1]). Indeed, Dennis, Scott and Laudet [2] have argued that it takes around five years before recovery can be considered to be ‘self-sustaining’. There is now a growing consensus that recovery is a multi-factorial and non-linear process, with the Betty Ford group defining addiction recovery as “voluntarily maintained lifestyle characterised by sobriety, personal health and citizenship” ([1] , p. 222]). The Betty Ford definition also differentiates between ‘early recovery’ (of up to one year), ‘sustained recovery’ (of between one and five years) and ‘stable recovery’ (of more than five years). A similar definition was developed by the UK Drug Policy Commission, suggesting the possibility of non-abstinent recovery, which defined recovery as “voluntarily sustained control over substance use which maximises health and wellbeing and participation in the rights, roles and responsibilities of society” ([3] , p. 6]).

Not only has recovery been difficult to capture, it is equally difficult to measure and this has led to the development of a metric for encapsulating recovery progress termed ‘recovery capital’, originally defined as ‘the sum total of one’s resources that can be brought to bear on the initiation and maintenance of substance misuse cessation’ ([4] , p. 1972]). Best and Laudet [5] have subsequently suggested that recovery capital can be split into three domains of assets – personal recovery capital (referring to the skills and capabilities the person possesses); social recovery capital (referring to the strength of associations to positive social networks) and community recovery capital (referring to the availability and accessibility of resources such as jobs and houses in the local community). This has spawned a growing interest in the measurement of recovery capital that has resulted in the development of the Assessment of Recovery Capital scale ([ARC [6];) based on the idea that recovery capital is not only something that can be measured, but it can be assessed at various moments as it changes over time. The REC-CAP study [7] demonstrated the idea of dynamism in recovery capital, but also the possibility of providing people in recovery and their navigators with the tools to monitor strengths and to use these strengths to support an ongoing recovery growth pathway. Yet, other tools and measures may offer ways of assessing recovery strengths and deficits in recovery.

Faces and Voices of Recovery (FAVOR), a US-based peer recovery organisation, commissioned an online survey of Life in Recovery (LiR), with recruitment also taking place online and through snowballing methods, targeting people who saw themselves at various stages of recovery. The aim was to be inclusive and to allow participants considerable discretion in their definition of ‘recovery’, and to recruit from a diverse range of treatment and community groups. In the initial study described in Laudet [8], 44 items representing experiences and measures of functioning in the domains ‘work’, ‘finances’, ‘legal status’, ‘family and social relations’, and ‘citizenship’ were supplemented with basic demographic questions and questions about recovery stage; each question was asked for when the person was “in active addiction” and again “since you entered recovery.” A total of 3228 surveys were completed and returned from US participants. The author concluded that “recovery from alcohol and drug problems is associated with dramatic improvements in all areas of life: healthier/better financial and family life, higher civic engagement, dramatic decreases in public health and safety risks, and significant increases in employment and work” ([8] , p. 3]).

This LiR method has subsequently been adapted for use in a range of other countries with McQuaid and colleagues [9] undertaking the first Life in Recovery survey in Canada and similar activities having already taken place in Australia [10] and the UK [11]. The same broad themes emerged – including marked improvements across the same five domains of work, finances, legal status, family and social relationships, and citizenship – but with sufficient local variations to suggest that recovery pathways are not insensitive to local and cultural contexts. A multi-country study (REC-PATH) using the LiR was recently set up to compare recovery experiences and pathways in three countries and to assess the role of contextual and societal factors [12].

Nonetheless, the LiR format has not yet been quantified, nor have summary measures been developed to examine changes in recovery deficits and recovery strengths during the recovery process. Therefore, the rationale for the current paper is to undertake a preliminary quantification of the LiR data to create summary scales that measure deficits and strengths and to assess how these indicators change across the recovery journey. The research questions addressed in this paper are:

RQ1: Can the LiR items be classified into recovery strengths and recovery barriers and can this be used to create overall strengths and barriers scores?

RQ2: Do these recovery capital measures differ across the stages of recovery (early, sustained and stable recovery) outlined in the Betty Ford Consensus Group document [1]?

RQ3: What predicts the total number of recovery resources an individual has in recovery?

## Methods

Best and colleagues [12] have outlined the design and methods for the REC-PATH study, a multi-site longitudinal study of (gender) differences in recovery pathways in the UK, Netherlands and Belgium, with an exclusive focus on illicit drug recovery. The screening instrument that was used to recruit to this study was the Life in Recovery survey (LiR) with only slight adaptations for the study and a Dutch/Flemish translation [13]. When this survey was launched online, a partnership was developed with the international Recovered Users Network (RUN) to translate the survey into a number of other European languages and to run the study in other countries. A version of the instrument adapted only to change the demographic characteristics was circulated through the Recovered Users Network (RUN), following a process of back-translation to ensure both consistency and that it was meaningful to potential participants. Distribution of the survey relied on support and participation of recovery organisations, therapeutic communities, communes and services providing various supports to people affected by drugs and those in recovery across Europe. The sample should be regarded as a network of these organisations. Member organisations of Recovered Users Network (RUN), the coordinators for their respected countries, have played the most significant role in coordinating data collection.

### Procedure

The survey has been translated into the local languages of the countries involved, namely Bosnian/Croatian/Serbian/Montenegrin, Swedish, Polish, Portuguese and Spanish, and it ran for four months, from January 18th, 2018 until June 12th, 2018. The survey was available online on the REC-PATH project platform: https://www.rec-path.co.uk/, as well as in hard copies and it was promoted regionally via organisations, social media, websites, TV shows and other partner agencies. The survey used the online platform Qualtrics. Participants could choose which language they wanted to complete the form in. Online information and consent preceded initiation of the questionnaire. For participants to complete the form, each item of each section required an endorsement or they would not be able to pass onto the next question. Consequently, only completed questionnaires were available on the online platform. Hard copies of the survey were made available for those who did not have access to or were not comfortable completing the online version. The aim (as with other LiR surveys) was to start from known recovery groups and then snowball out to a more diverse group of potential participants. Online completion was encouraged as often as possible and only completed hard copies were entered into the database. Thus, no missing data had to be managed in the analysis.

### Development of the strengths and barriers recovery scale (SABRS)

The aim of the study was to ‘translate’ as many of the items of the LiR measure into a new scale consisting of positive and negative experiences and events that could be characterised as positive and negative recovery capital.

From the original set of 44 items in the US LIR, two had been removed for the Australian and UK versions as they did not apply (‘did not have health insurance’ and ‘lost right to vote’). For the current scale, items were removed if they could only apply based on something else happening – thus, a professional licence can only be restored if you have had one in the first place. Similarly, the item ‘had your driving license restored’ rests on two prior conditions – first that the person has a driving license and second, that it has been revoked. Similarly, losing or having a professional registration restored is contingent on the person having a professional registration in the first place. This resulted in a total of 32 items included in the final scale (15 strength items and 17 barrier items) and 10 items excluded (as shown in Tables 1 and 2). All items had binary (yes/no) response options.

**Table 1:** Final set of included items in the Strengths And Barriers Recovery Scale (SABRS) (*n* = 32)

Recovery strength items	Recovery Barrier items
- Exercise regularly - Have a GP - Have regular dental checks - Have good nutrition - Take care of your health - Maintain a driving license - Maintain a bank account - Able to pay your bills - Maintain stable housing - Remain in steady employment - Further your education or training - Start your own business - Participate in family life - Plan for the future - Volunteer	- Have untreated emotional or mental health problems - Make regular visits to the emergency room - Regular use of health services - Smoke - Have your drivers’ license revoked - Drive under the influence of alcohol or drugs - Damage property - Been arrested - Been charged with a criminal offence - Been to prison - Have bad debts - Were unable to pay the bills - Regularly missed school or work - Dropped out of school or college - Fired or suspended from work - Lose custody of children - Experience family violence

**Table 2:** Excluded items from the Life In Recovery Scale

- Get your driver’s license back - Complete a term of conditional release or parole - Have credit restored - Owe back taxes - Pay back taxes - Pay your taxes on time - Receive good job evaluations - Lose your professional license - Have your professional license restored - Regain custody of your children	

The next stage was to separate items into strength and barrier items. All five domains of the LiR – work, finances, legal status, family and social relationships, and citizenship – contained a combination of strength and barrier items and the total remaining items were classified as 15 recovery strength and 17 recovery barrier items. All of these individual items were endorsed if they applied and so were coded as 0 or 1. This meant that the strengths scale ranged from 0 to 15 and the barriers scale from 0 to 17. There were four totals then calculated:

- Recovery strengths in active addiction (0–15)
- Recovery barriers in active addiction (0–17)
- Recovery strengths in recovery (0–15)
- Recovery barriers in recovery (0–17)

### Sample

Table 3 provides a breakdown of the sample (*n* = 480) for each country of residence – this was recorded rather than nationality. Although the number is small from some of the participating countries, for ethical reasons we did not want to exclude any participant who had successfully completed the survey. The mean age of the sample was 37.6 years (SD = 9.3 years); 68.3% of the sample described themselves as male; 31.0% as female and 0.6% as of other gender alignments. In terms of education, 4.4% did not complete primary school, 17.1% achieved only primary school education, 52.2% achieved secondary education and 24.4% achieved a higher education qualification. In terms of relationships, 41.5% were single and never married, 41.7% were married or co-habiting, 12.7% were separated or divorced. 1.7% were widowed and 2.2% described their relationship status as ‘other’. Thirty-nine per cent of the sample reported that they had dependent children. Just over half of the participants (54.7%) were from the Balkan region.

**Table 3:** Country of residence of participants (*n* = 480)

		Frequency	Percent	Gender - % female	Age (mean, SD)
Valid	Bosnia & Herzegovina	72	15.0	29.2%	35.4 (8.1)
	Serbia	123	25.6	26.0%	36.6 (5.9)
	Croatia	53	11.0	35.8%	39.7 (6.2)
	Montenegro	15	3.1	6.7%	40.1 (5.9)
	Poland	79	16.5	38.0%	29.0 (7.5)
	Portugal	6	1.3	33.3%	40.3 (7.4)
	Spain	60	12.5	18.3%	45.9 (8.5)
	Sweden	44	9.2	61.4%	41.9 (12.9)
	Other (Please specify)	28	5.8	21.4%	40.7 (7.9)
	Total	480	100.0	100.0	

### Analysis

The primary aim of the paper is to examine differences in the newly developed measure of recovery strengths and barriers (SABRS) between active addiction and current experiences in recovery, but also to assess if this varies as a function of the stage of recovery that the respondent is in. Therefore, the initial analysis is a repeated measures t-test comparing strengths and deficits in active addiction and recovery. The second stage of the analysis compares recovery strengths and barriers (both in active addiction and recovery) by stage of the recovery journey (1).

The final analysis will attempt to predict the total number of recovery strengths in recovery to address research question 3. This involved undertaking a linear regression with backwards elimination using all key demographic measures (age, gender), addiction career factors (age of first and last use, and length of time in recovery) and both barriers and strengths while in active addiction and ongoing barriers while in recovery. As duration of recovery was used as a continuous variable, recovery stage was excluded. No bivariate correlation exceeded 0.7 and so collinearity was not a reason for item exclusion.

## Results

### Overall patterns of strengths, barriers and change

Table 4 shows a clear pattern of reduced recovery barriers (from a mean of 8.2 to 2.4 out of a possible 17) and increased recovery assets (from a mean of 4.1 to 9.2 out of a possible 15) that occurs in the transition from active addiction to recovery. In active addiction, it is not the case that the person does not have recovery capital, but that it is swamped by the number of barriers to change the experience. Likewise, the transition to recovery does not mean the complete elimination of barriers, but by this point they are surpassed by assets and strengths the individual can call on to support their recovery journey.

**Table 4:** Recovery barriers and recovery strengths in active addiction and in recovery

	Active Addiction	Recovery	T, significance
Number of recovery Strengths	4.1 (SD = 2.6)	9.2 (SD = 3.7)	26.51, *p* < 0.001
Number of recovery Barriers	8.2 (SD = 3.4)	2.4 (SD = 2.4)	30.92, *p* < 0.001

The first key finding is that people in active addiction do have recovery assets and strengths, and it is also the case that those who are in recovery have ongoing deficits. Addiction is not the absence of strengths in the same way that recovery is not the absence of challenges and life problems. Nonetheless, there are hugely significant changes with recovery barriers diminishing and recovery strengths growing across recovery stages, but it is also interesting to note that there is a positive association between strengths in addiction and strengths in recovery (r = 0.14, *p* < 0.01), while there is no significant link between the number of barriers in active addiction and in recovery. For each person, we were then able to create two scores: 1. Change in recovery strengths (from active addiction to recovery); and 2. Change in recovery barriers (from active addiction to recovery). Thus, across the sample there was reduction of 5.79 (SD = 3.97) in barriers to recovery and an increase in strengths of 5.10 (SD = 4.22) from active addiction to recovery. There was a very strong inverse association (r = − 0.45, *p* < 0.001) between the reduction in deficits and the growth in strengths.

### Recovery strengths and barriers by stage of recovery

The next step of the analysis looks at each of these factors by stage of recovery as shown in Table 5.

**Table 5:** Recovery strengths, barriers and change by recovery stage

Mean number of … … …	Early recovery mean (SD)	Sustained recovery mean (SD)	Stable recovery mean (SD)	F, significance
Recovery strengths in active addiction	4.7 (2.8)	3.9 (2.6)	3.8 (2.4)	6.41, *p* < 0.01
Recovery barriers in active addiction	7.2 (3.4)	8.6 (3.4)	8.8 (3.1)	10.69, *p* < 0.001
Recovery strengths in recovery	7.8 (3.6)	9.0 (3.6)	10.7 (3.2)	28.52, *p* < 0.001
Recovery barriers in recovery	2.6 (2.3)	2.6 (2.5)	2.2 (2.4)	1.73, *p* = 0.18
Strengths change	3.1 (3.6)	5.1 (4.1)	6.9 (4.0)	39.56, *p* < 0.001
Barriers change	−4.7 (3.9)	−6.0 (4.0)	−6.6 (3.8)	10.07, *p* < 0.001

Recovery strengths show consistent differences between people at different stages in the recovery journey and recovery barriers also show considerable variability from active addiction to recovery. However, there are no significant stepwise reductions in ongoing barriers to recovery between people at different stages of the recovery journey. In other words, for many people in stable recovery, there are ongoing barriers to their recovery wellbeing, that do not differ markedly between those in early, sustained or stable recovery. While we can infer that strengths accrue with recovery time, barriers do not appear to diminish in the same way. It is also interesting to note that people who are in early recovery perceived themselves to have more strengths and fewer barriers in active addiction, in comparison to those in the later stages of their recovery journeys.

### Recovery strengths and barriers by gender

As outlined in Table 6, there are some notable differences between men and women in their barriers and strengths to recovery and in the process of change over time. While men reported significantly more recovery strengths during their time in active addiction, this situation is reversed with women reporting significantly more strengths in recovery, and therefore a much clearer presumption of growth of recovery assets in the period in between. There is a much less striking pattern for barriers with men reporting slightly more barriers both in addiction and in recovery and with no gender difference in the reduction in recovery barriers in the time in between.

**Table 6:** Gender differences in recovery barriers and strengths

Mean number of … .	Male (***n*** = 328)	Female (***n*** = 149)	T, df, significance
**Strengths in active addiction**	4.3 (SD = 2.6)	3.7 (SD = 2.7)	2.37, 475, *p* < 0.05
**Barriers in active addiction**	8.4 (SD = 3.3)	7.9 (SD = 3.4)	1.50, 475, *p* = 0.13
**Strengths in recovery**	8.8 (SD = 3.8)	10.3 (SD = 3.1)	4.80, 346.5, *p* < 0.001
**Barriers in recovery**	2.7 (SD = 2.6)	2.0 (SD = 1.9)	3.11, 385.3, *p* < 0.001

- The final analysis attempted to predict the total number of recovery assets in recovery using the following factors as independent variables: age, gender, length of recovery, age of first drug use, age of last drug use, recovery strengths in active addiction, recovery barriers in active addiction and recovery barriers in recovery

The final model was highly significant (F = 28.58, *p* < 0.001), predicting 31.8% of the variance of recovery strengths for individuals in recovery, with only age of first use as a non-significant predictor in the final model (see Table 7). The largest effects were found for current recovery barriers, for both strengths and barriers in active addiction and for gender. In other words, how many recovery strengths a person has in recovery is predicted by their age and gender, by their duration of recovery and time since last use, but crucially for the current analysis by the number of strengths and barriers they had in active addiction and by the number of barriers they continue to have in recovery.

**Table 7:** Factors retained in the backwards elimination regression predicting recovery strengths in recovery

Model			95% CI for B Unstandardized Coefficients		Standardized Coefficients	t	Sig.
		B		Std. Error	Beta		
1	(Constant)	4.096	2.00–6.19	1.067		3.837	*P* < .001
	Active addiction deficits	.310	0.24–0.46	.043	.285	7.146	*P* < .001
	Active addiction strengths	.350	0.22–0.39	.054	.254	6.478	*P* < .001
	Recovery deficits	−.430	−0.54 - -0.31	.059	−.284	−7.345	*P* < .001
	What is your age?	.105	0.06–0.15	.023	.266	4.620	*P* < .001
	What is your gender?	1.203	0.59–1.77	.298	.159	4.033	*P* < .001
	How old were you when you first used any illicit drug?	−.062	−0.14 – 0.01	.039	−.065	−1.603	*P* = .110
	How old were you when you last used any illicit drug?	−.087	−0.13 - -0.14	.021	−.237	−4.204	*P* < .001
	Total Recovery Duration in Months	.006	0.02–0.12	.002	.116	2.416	.016

a. Dependent Variable: recovery strengths at follow-up

## Discussion

The LiR was used to measure strengths and barriers in active addiction and recovery among a diverse population of 480 persons in illicit drug recovery from various European countries. One of the key findings of this study is that, while we replicate previous research showing that the recovery journey involves the accretion of assets and the reduction in barriers [7], the average person does have recovery assets at the peak of their addiction, and they continue to have barriers to capital growth in their recovery. What is also apparent is that the extensity of recovery does predict a continuing growth of resources and strengths as would be predicted by Dennis and colleagues [2], but there is much less evidence that the residual barriers to recovery are overcome quite so readily. This may be because some of them – such as residual emotional and mental health problems may persist in spite of recovery extensity and longevity – while others may simply be the complications of everyday life that persist irrespective of recovery status [13].

The study is also innovative in that it uses an established international instrument for monitoring recovery pathways (the Life In Recovery survey) and edits this down to create an index of recovery capital called the Strengths And Barriers Recovery Scale (SABRS). The scale creates both total strengths scores and total barriers scores, both for the time of completion (in recovery) and for ‘at the peak of active addiction’. While susceptible to self-serving and recall barriers (see the limitations section below), this provides a recovery capital measure that charts ‘distance travelled’ as well as the barriers yet to be overcome in a recovery pathway. This scale offers the first attempt at quantification of the Life In Recovery survey that can be enhanced in future studies and with further samples.

The current data would also suggest that recovery has a gendered dimension. Wincup [14] has argued that women’s substance use is qualitatively different from men’s, yet drug and alcohol policy has glossed over the role gender might play in the processes of addiction and recovery [14, 15]. Research on women’s drug use indicates higher rates of mental health problems, experiences of physical and sexual abuse within childhood and adulthood, and involvement in sex work [16, 17]. Our findings provide a new perspective on these life-course issues in relation to substance addiction and gender – in active addiction men have both slightly more strengths and slightly more barriers, yet this situation is reversed in recovery. Not only do female participants report a greater growth in strengths to recovery, they also typically report more strengths in recovery than do their male counterparts (with no difference in the reduction of deficits). Although the reasons for this are not clear, this suggests that women who do achieve and sustain recovery are skilled at developing a diverse range of skills, having on average 10 of the 15 strengths or resources measured in the Life in Recovery scale.

Further, this gender difference is also maintained in the regression of overall recovery strengths. In addition to things that have been established in the evidence base such as longer duration of recovery, earlier age of cessation of drug use, both being female and being older were associated with greater positive recovery capital [2]. Furthermore, recovery strengths are inversely linked to the number of ongoing recovery barriers people experience, but are also linked to the number of strengths and resources a person had in active addiction. This may have implications for the extent of loss or exclusion caused by addiction but requires considerably more analyses and replication with a larger sample, as does the role of deficits in active addiction.

This study has a number of limitations common to the Life in Recovery model, discussed in Best and Edwards [18]. We have no control over the sampling and no knowledge of the overall population from which the sample are drawn. Thus, we can make no conclusions about representativeness and there are no attempts at objectifying or verifying the self-reported data either for the current time ‘in recovery’ or for experiences at the peak of addiction, and both addiction and recovery status are entirely reliant on self-report. It is possible that we are reporting a ‘halo’ effect, where the adverse effects of addiction are over-stated and the benefits of recovery equally exaggerated. Also, the scale used in this study has not been subjected to psychometric examination and is unlikely to be a comprehensive measure of all of the strengths and barriers to recovery that occur. Finally, Ashford and colleagues [19] have argued that recovery should be considered an organising concept, and here we have relied on its self-ascription for engagement in the study. In other words, people who do not consider themselves as ‘recovered’ or ‘in recovery’ are extremely unlikely to have completed it.

Nonetheless, the current paper is a significant enhancement to the existing body of Life in Recovery research by offering a mechanism for quantifying key aspects of positive and negative recovery capital to create a scalable measure that can assess both current situation and change. The paper challenges some of the existing assumptions about gender patterns in addiction and recovery and suggests a different change trajectory for women. The magnitude of effects around change and in gender differences do suggest clear differences that need to be tested and replicated in further studies. The study also provides further evidence around residual barriers and obstacles to recovery and understanding how these can be addressed and how they can be overcome through the development of strengths will be a critical aim for future recovery science. However, this work is ongoing and our intention is to run future iterations of the LiR survey that will increase power and will allow us both to examine cultural variations in recovery pathways and more in-depth analyses of country to country variations in both samples and responses, building on, for example, the REC-PATH study [12].

## Conclusion

The Life In Recovery survey has now been used across a number of countries to demonstrate the population level changes that occur when individuals transition from active addiction to recovery, and furthermore how these build across the three stages of early to sustained to stable recovery. However, no prior attempt has been made to create summary measures of recovery barriers and strengths, or the resulting capacity to assess the extent of change at the individual level. The SABRS measure reported here provides a way of summing data that allows individual level measurement of recovery capital and its potential deployment in peer and professional settings to support recovery growth.

## Data Availability

All data and materials will be made available on request from the ERANID project team.

## Abbreviations

ARC: Assessment of Recovery Capital
FAVOR: Faces And Voices Of Recovery
LiR: Life in Recovery
REC-PATH: Recovery Pathways (European Research Study)
RUN: Recovered Users Network
SABRS: Strengths And Barriers Recovery Scale
SD: Standard Deviation
